# Keeping the Balance: GABA_B_ Receptors in the Developing Brain and Beyond

**DOI:** 10.3390/brainsci12040419

**Published:** 2022-03-22

**Authors:** Davide Bassetti

**Affiliations:** Institute of Physiology, University Medical Center, Johannes Gutenberg University, 55218 Mainz, Germany; dbassett@uni-mainz.de

**Keywords:** GABA receptors, GABA_B_ receptors, development

## Abstract

The main neurotransmitter in the brain responsible for the inhibition of neuronal activity is γ-aminobutyric acid (GABA). It plays a crucial role in circuit formation during development, both via its primary effects as a neurotransmitter and also as a trophic factor. The GABA_B_ receptors (GABA_B_Rs) are G protein-coupled metabotropic receptors; on one hand, they can influence proliferation and migration; and, on the other, they can inhibit cells by modulating the function of K^+^ and Ca^2+^ channels, doing so on a slower time scale and with a longer-lasting effect compared to ionotropic GABA_A_ receptors. GABA_B_Rs are expressed pre- and post-synaptically, at both glutamatergic and GABAergic terminals, thus being able to shape neuronal activity, plasticity, and the balance between excitatory and inhibitory synaptic transmission in response to varying levels of extracellular GABA concentration. Furthermore, given their subunit composition and their ability to form complexes with several associated proteins, GABA_B_Rs display heterogeneity with regard to their function, which makes them a promising target for pharmacological interventions. This review will describe (i) the latest results concerning GABA_B_Rs/GABA_B_R-complex structures, their function, and the developmental time course of their appearance and functional integration in the brain, (ii) their involvement in manifestation of various pathophysiological conditions, and (iii) the current status of preclinical and clinical studies involving GABA_B_R-targeting drugs.

## 1. Introduction

γ-aminobutyric acid (GABA) is the main neurotransmitter in the central nervous system that in adult age mediates the inhibition of neurons by acting on two classes of receptors. The activation of ionotropic receptors—GABA_A_ and GABA_C_—leads to a flux of chloride ions in accordance with the driving force. Metabotropic GABA_B_ receptors (GABA_B_Rs) are instead associated with Gi proteins, which lead to the inhibition of neuronal activity following activation. 

During early development, many processes related to circuit formation must be dynamically fine-tuned and coordinated [1]. In this period, the inhibitory system undergoes prominent changes. The maturation of the inhibitory system is in fact a dynamic process that is crucial for correct brain functioning [2]. GABAergic neurons in the neocortex originate from the subpallium [3] and proceed to invade the pallium. Here, they shape the formation of—and integrate in—the existing circuit and, in rodents, they reach a mature state by postnatal day 30 [4,5]. Interneurons have an active role in development by influencing network activity [6,7], but their contribution is not limited to this aspect. In fact, GABA not only has an effect as a neurotransmitter, it also has a neurotrophic function on cell growth and network formation [8,9]. 

The role of GABA_A_ receptors (GABA_A_Rs) in development has been revealed by many studies, as summarized by excellent reviews on this topic [2,10]. However, GABA_B_Rs have remained, in comparison, less investigated in this context despite the clear involvement they have in several processes such as learning and memory [11] or the shaping of neuronal circuits [12]. The dynamic adjustment of these receptors during development and how alterations in their function can affect brain growth are becoming emergent topics [13,14]. Recent technical advancements allow a detailed understanding of the structure of GABA_B_Rs. This will enable a more precise pharmacological modulation of GABA_B_Rs with the possibility of investigating them at a much deeper level [15]. This review article covers recent discoveries concerning GABA_B_Rs, their role during development, and current as well as potential future therapeutic applications.

## 2. GABA_B_Rs: Structure and Function

**Figure 1:**
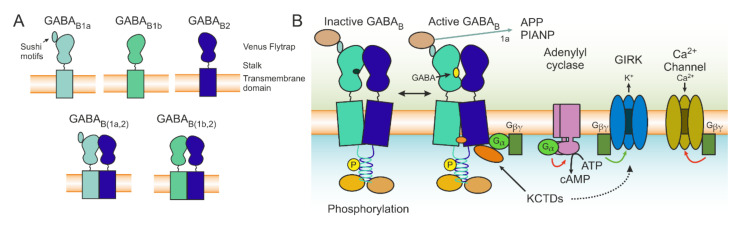
Composition and main functions of GABA_B_Rs. (**A**) Schematic representation of the subunit structure (top row) and of heterodimers (bottom row). (**B**) Diagram illustrating activation of a heterodimer, including a G protein and downstream effectors. The G protein subunits can inhibit the activity of adenylyl cyclase, thus reducing the levels of cAMP and of Ca^2+^ channels. Another consequence is the activation of GIRKs, which can be modulated by KCTDs. Associated proteins such as APP and PIANP are also included in the scheme (for details, see text).

The inhibitory effect of GABA in the CNS has been observed and described for more than 50 years [16,17]. Dr. Norman Bowery was the first to describe a class of GABA receptors that could reduce the release of neurotransmitters and that were not sensitive to isoguvacine or bicuculline, thus distinguishing them from the established GABA receptor, naming the newly found GABA_B_ and the previously known GABA_A_ [18]. A functional GABA_B_R is constituted of an obligatory heterodimer of the GABA_B1_ and GABA_B2_ subunits; GABA_B1_ is required for ligand-binding and GABA_B2_ is necessary for interactions with G proteins as well as increasing the affinity of GABA_B1_ to GABA [19,20]. The common domains between the two subunits are a C-terminal intracellular domain, a heptahelical transmembrane domain, and a Venus flytrap domain on the extracellular side that is connected by a stalk (Figure 1A). The GABA_B1_ receptor contains an endoplasmic reticulum (ER) retention tag that is masked following interaction with GABA_B2_, thus allowing for correct receptor transport [21]. Although GABA_B1_ possesses several splice variants, the most common are GABA_B1a_ and GABA_B1b_. The main structural difference between the two is the presence of two sushi domains on GABA_B1a_ [22], which influences the transport of the receptor and thus makes GABA_B(1a,2)_ more stable in the pre-synaptic site and in the dendritic compartments, while GABA_B(1b,2)_ is responsible for post-synaptic inhibition in spines (Figure 1A) [23].

**Figure 2:**
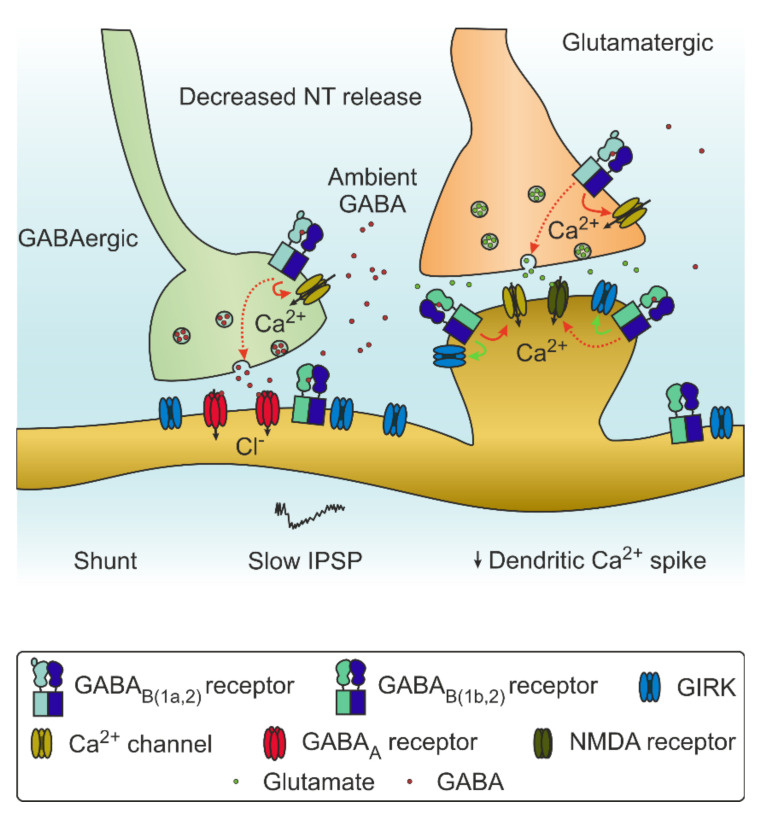
Functions of GABA_B_Rs in modulation of synaptic transmission. GABA_B_Rs are expressed pre-synaptically at both GABAergic (left, green) and glutamatergic (right, orange) synapses, where they can inhibit vesicle fusion and neurotransmitter release through inhibition of Ca^2+^ channels. They are also present post-synaptically, and they influence GIRK as well as the NMDA receptor function. Activation of GABA_B_Rs on the post-synaptic side leads to slow GIRK-channel-mediated IPSP and shunting inhibition, which can consequently inhibit dendritic calcium spike propagation. For details, see text.

Recently, the structure of GABA_B_Rs in different conformations have been described using cryo-electron microscopy (unbound [24,25,26,27,28] and bound to agonist, modulators, and G protein [29]), allowing a greater understanding of their assembly and paving the way to the design of more advanced pharmacological tools for their modulation (structural results are reviewed in [30]). Activation by orthosteric binding results in a conformational shift which allows interaction with a heterotrimeric G protein. Upon binding, the G protein dissociates into Gα and Gβγ subunits. The Gα subunits most often associated to GABA_B_Rs are Gα_i_ and Gα_o_. The Gα_i/o_ subunit binds to adenylyl cyclase (AC), diminishing its activity and subsequently the levels of cAMP. Reduced cAMP levels in turn lead to a reduced probability of pre-synaptic neurotransmitter release. On the post-synaptic side, the protein kinase A pathway is also influenced by reduced AC activity, which results in decreased NMDAR conductance. The Gβγ fragment can also diminish vesicle fusion and the release of a neurotransmitter via inhibition of voltage-gated calcium channels. On the post-synaptic side, GABA_B_Rs activate G protein-coupled inward rectifying K^+^ channels (GIRK), which causes a hyperpolarizing slow inhibitory post-synaptic current that transiently inhibits the cell. The resulting increase in conductance contributes to the shunting effect on ongoing post-synaptic currents and shunts the backpropagation of the dendritic calcium spike [31] (Figure 1B and Figure 2).

Recent reports suggest that in cerebellar granule neuron cultures, GABA_B_Rs can influence synaptic strength and even provide an antiapoptotic effect, as they do not only couple to Gα_i_ and Gαo, but they can also activate the G_α13_ proteins. This action is performed at a much slower speed than those that are classical [32]. During development, GABA_B_Rs can also associate with Gα_q_, which enhances voltage-dependent calcium currents without Gα_i/o_ [33]. Moreover, GABA_B_Rs can also present non-canonical effects, such as in the nucleus accumbens, where GABA_B_Rs activation likely inhibits glutamatergic pre-synaptic terminals by inhibiting the assembly of SNARE complexes [34].

GABA_B_Rs possess a multitude of regulatory mechanisms that can affect their functionality, for example heterodimers can dynamically associate to form oligomers [35] in which the ligand affinity decreases by interaction with neighboring receptors [36]. Many interacting proteins that can associate with GABA_B_Rs and alter their properties have been discovered. The K^+^ channel tetramerization domain containing proteins (KCTDs) KCTD8, KCTD12, KCTD12b, and KCTD16 are examples of auxiliary subunits of GABA_B_Rs [37]. The association of KCTDs with GABA_B_Rs influences the kinetics of GABA_B_R-mediated responses, allowing for a faster interaction and consequently decreasing the rise time. In addition, KCTD12 can compete with Gβγ, thus inducing desensitization [38,39]. The KCTD 8 and the KCTD16 can prevent such desensitization [40], and different KCTDs can form hetero-oligomers with mixed effects on both the desensitization and the deactivation of K^+^ currents. The KCTDs therefore provide the possibility of precise fine-tuning of kinetics and the GABA_B_R function [41], and this tuning can in turn be regulated depending on brain region and age. The KCTDs in fact display varying degrees of region and layer specificity, as well as temporal changes in expression [42]. A lack of KCTD12 or KCTD16 in mice can lead to alterations in fear processing and emotivity, thus further highlighting their importance for a correct regulation of GABA_B_Rs [43,44].

However, the possibilities of GABA_B_R regulation are much larger. By using high-resolution proteomics, Schwenk and colleagues described a large amount of interacting proteins that participate in the formation of GABA_B_R complexes, including effector proteins [45]. The exact composition of the complex can explain the heterogeneity of functions and constitute a promising target for future drug design (for review, see [46]). Between the described interacting proteins in GABA_B_R complexes, the authors could find hyperpolarization activated cyclic nucleotide channel 2 (HCN2), which interacts with the complex through KCTD16. This interaction was shown to shorten the duration of IPSPs in dopaminergic neurons [45]. Furthermore, GABA_B1_Rs can inhibit the sensitization of transient receptor potential vanilloid 1 (TRPV1) channels in a G protein- and GABA_B2_-independent fashion [47]. Some components of the complex bind through the sushi domain of GABA_B__1a_, including the β-amyloid precursor protein (APP, the precursor of β-amyloid peptides), PILRα-associated neural protein (PIANP), and adherence-junction associated protein 1 (AJAP-1), and they can influence the trafficking of GABA_B_Rs. These proteins have particular pathophysiological relevance (described in the following sections) [45]. Another factor to consider is the number of GABA_B_Rs on the cell surface. In fact, these receptors undergo constitutive endocytosis, which can be followed by either degradation or recycling to the membrane. Sustained glutamate-induced calcium influx can quickly and selectively diminish the rate of recycling, leading to a reduced GABABR-mediated inhibition [48]. Furthermore, additional mechanisms such as phosphorylation or ubiquitination have been shown to influence GABA_B_R functions (for a review, see [49]). 

Despite the relatively restricted number of subunits and isoforms, the family of functional GABA_B_Rs presents an extensive level of variety, which is granted by the formation of complexes, the oligomeric state, the phosphorylation state, and a large number of interacting proteins. 

## 3. Spatial and Temporal Localization of GABA_B_Rs

In both rodent and in human adult brains, GABA_B_Rs are widely distributed across many brain areas, showing a distribution similar to that of GABA_A_Rs, albeit with a smaller number [50,51]. In rodents, the pharmacology and distribution of GABA_B_Rs varies during development in a region-specific manner [52,53,54]. In the neocortex, both subunits are expressed starting from embryonic stages [53]. During the first two postnatal weeks, the distribution of both subunits across the rodent brain varies almost independently, reaching a pattern of expression comparable to adults at around P20, with a general decrease thereafter [55]. The GABA_B1_ subunit appears near birth, mostly in superficial layer neurons with a Cajal–Retzius morphology. The GABA_B2_ subunit is also expressed early during development in the neocortex, especially in superficial layers, and it then becomes more uniformly distributed after P15 [53]. The GABA_B1a_ and GABA_B1b_ isoforms are also differentially regulated during development. GABA_B1a_ is the predominant isoform at birth and it decreases over time, reaching adult levels after the end of the first postnatal month. On the other hand, the GABA_B1b_ level at P0 is circa 50% of the adult level, and it undergoes a strong increase which peaks near P10 and decreases subsequently [56]. Near P10, the GABA_B1_ subunits in pyramidal neurons of the superficial cortical layers relocate from the soma and the dendrites to a more uniform spread across the whole cell membrane [53]. 

The distribution of GABA_B_Rs in the developing and the mature hippocampus has also been described in detail [54,57]; GABA_B_Rs are expressed at embryonic stages, they assemble both pre- and post-synaptically, and they show developmental regulation [58]. The distribution of GABA_B1_ and GIRKs is not homogeneous across cells, but rather arranged in a lamina-specific manner. The resulting GABA_B_R-mediated potassium conductance is limited by the availability of both proteins [59]. Moreover, in the dentate gyrus, optogenetic stimulation of specific subtypes of interneurons demonstrated how the single stimulus GABA_B_-evoked GIRK response strength varies between different types of interneurons [60]. 

In summary, GABA_B_Rs expression is regulated throughout development in the cortex and in the hippocampus, and their function can be further modulated via the control of the expression of effectors as well as auxiliary and interacting proteins. Indeed, in addition to the variation of GABA_B_R subunit expression during development, it is also important to take into account how the interacting proteins in the receptor complex tend to display developmental variations [45]. The fact that both subunits of GABA_B_Rs are present so early during development, before the establishment of mature synaptic transmission, suggests they might play a part in developmental mechanisms.

## 4. Developmental Functions

While GABA regulates fundamental steps in CNS development–including cell migration during cortex formation [61,62], cell maturation, and network development [13,63]—more recently, the role of GABA on neurogenesis has been described, mostly with a focus on GABA_A_ receptors [64]. However, GABA_B_Rs have also been shown to be able to influence adult neurogenesis in the hippocampus. In their niche, neural stem cells (NSCs) express functional GABA_B_Rs, which suppress their proliferation and their differentiation. Their effect is opposite to that of GABA_A_Rs, whose activation promotes differentiation and integration in the circuit. This suggests that both receptors may work together and balance each other antagonistically [64,65]. It is important to notice that adult born granule cells lack GIRKs, which appear only subsequently, after approximately three weeks of maturation [60]. 

In a recent study, with the aim of investigating the role of GABA receptors in early neurogenesis, GABA_A_- and GABA_B_Rs were transiently blocked between P6 and P11. The blockade of GABA_B_Rs, but not of GABA_A_Rs, reduced the number of proliferating NSCs and the intermediate progenitor cells in the dentate gyrus. Furthermore, GABA_B_R blockade caused a decreased expression of neurotrophins which are associated with synaptic plasticity, such as brain-derived neurotrophic factor (BDNF), nerve growth factor (NGF), and neurotrophin 3 (NT-3) [66]. Contrarily, GABA_B_R activation can trigger BDNF release and promote inhibitory synaptogenesis in the newborn hippocampus [67], which can affect the development of GABAergic transmission [68].

In contrast to neurotrophic factors, GABA_B_Rs can interact directly with transcription factors such as activating transcription factor 4 (ATF4), which contributes to synaptic plasticity [69]. This interaction undergoes changes in efficiency during postnatal development and it is shared by neurons and glial cells [70]. Interestingly, ATF4 itself can have effects over longer time scales as a regulator of GABA_B_Rs trafficking, by acting on GABA_B1_ subunits and promoting surface exposure [71]. 

GABA_B_Rs also play a developmental role in the framework of transient cellular population. Cajal-Retzius cells are a transient cellular population which are present at embryonal stages [72,73]. They disappear towards the end of the second postnatal week and play an important role in circuit formation and correct lamination [74]. Between P5 and P7, GABA_B_Rs, together with glial transporters, constitute an important feedback mechanism for controlling the excitability of those cells [75]. Cajal-Retzius cells are one of the sources of Reelin protein, which is released extracellularly and guides cellular migration. In a recent study, Reelin was found capable of modulating the amount of both GABA_B1_ and GABA_B2_ on the cell surface. Furthermore, agonist and antagonist treatment of GABA_B_Rs in the absence of Reelin had no effect on the presynaptic side [76]. These results highlight the tight connection between GABA_B_Rs and Reelin, which is not only a key player in cell migration, but is also receiving increasing attention for control of synaptic formation and function [77]. 

The metabotropic GABA_B_ receptors, GABA_B_Rs, are not only expressed in neurons, but also in glial cells and their regulation has a developmental aspect. The importance of the role of astrocytes in the regulation of network activity is gaining growing attention [78]. Astrocytes communicate with GABAergic neurons, and they contribute to the regulation of synaptic transmission [79]. The activation of astrocytic GABA_B_Rs triggers a calcium transient via Ca^2+^ release from intracellular stores. In contrast to the calcium transients evoked by astrocytic GABA_A_R activation, GABA_B_R-evoked responses show a change during development in hippocampal astrocytes. At P3 and P33 the percentage of cells showing such responses was 10%, but between P11 and P15 it was instead 60% [80]. In a similar way, neocortical astrocytes also respond to GABA_B_R activation with calcium oscillations, albeit with only a slight decrease in the number of responding cells in slices from older animals [81]. Astrocytes, following activation with GABA, release glutamate that influences the activity of neighboring pyramidal neurons via the induction of slow inward currents [82]. Moreover, astrocytic GABA_B_Rs in the mouse hippocampus have been proposed to control the response to behavioral challenge through the regulation of the astrocytic release of BDNF [83].

Myelination in both the central nervous system and the peripheral nervous system is also influenced by GABA_B_Rs [84]. In fact, GABA_B_R activation has a stimulating effect on the differentiation of oligodendroglial cells and it can boost the expression of myelin-related protein expression [85]. Transient GABA_B_Rs blockade between P6 and P11 can decrease the level of myelin basic protein and affect the proliferation of oligodendroglial cells in vivo [86].

Furthermore, the organization of the inhibitory circuit during development in the first two postnatal weeks requires the presence of GABA_B_Rs in microglial cells. The knockdown of GABA_B1_ selectively in microglial cells led to a significant increase of inhibitory synapses originating from parvalbumin-positive (PV) interneurons onto pyramidal cells but no changes in excitatory synapses, with a consequent decrease in the ratio between the excitatory and the inhibitory post-synaptic current frequency at P30 [87]. Interestingly, the same animals at P60 display an almost reversed pattern, with a reduction in inhibitory synapses and no changes in the excitatory system, presumably via compensatory mechanisms. A lack of correct microglial-dependent synaptic organization led to a slight reduction in exploratory behavior at P30 and hyperactivity in P60 animals [87].

To sum up, our understanding of the role of GABA_B_Rs during development is becoming increasingly multifaceted and rich. They are involved in a variety of functions, from neurogenesis (both in adult age and during development) to migration, and they include transient cell types and glial cells.

## 5. Crosstalk with GABA_A_Rs and Early Activity Patterns

Other than the aforementioned neurotrophic or activity-independent role, GABA_B_Rs are also in a central position to exert control on the network excitation level; for their role in synaptic transmission, assembly in heterodimers is required. In both the cortex and the hippocampus, the pre-synaptic and the post-synaptic components develop at a separate pace. Indeed, post-synaptic GABA_B_R-mediated currents only appear in the second postnatal week [88,89]. On the other hand, functional activation of pre-synaptic GABA_B_Rs has been reported much earlier, at the end of the first postnatal week in the cortex and the CA1 region of the hippocampus [90,91], and even earlier (i.e., at birth) in the CA3 region [92]. 

The GABA_B_Rs display different mechanisms of crosstalk with other neurotransmitter systems including glutamate receptors [93] and GABA_A_Rs. For example, GABA_B_Rs activation is able to influence the decay of GABA_A_R-mediated currents as well as the mIPSC frequency [94]. This crosstalk is particularly relevant during early life. The development of the inhibitory system in the postnatal period is in fact a complex process [95] that includes strong changes in the function of GABA. In rodents, immature neurons regulate the intracellular chloride concentration mostly via the Na^+^-K^+^-Cl^-^ cotransporter isoform 1 (NKCC1), which leads to a steady state higher chloride concentration. Therefore, the opening of GABA_A_ receptors can result in an efflux of chloride ions, effectively depolarizing the cell. During development, the K-Cl cotransporter isoform 2 (KCC2) expression increases, reducing the intracellular Cl concentration to mature levels and thus influencing the GABA current reversing potential [6].

GABA_B_Rs associate with KCC2 in protein complexes, and they are therefore able to influence neuronal chloride regulation. Indeed, activation of GABA_B_Rs can reduce the efficacy of KCC2 as well as their surface expression. This mechanism allows elevated levels of extracellular GABA to influence the effect of the neurotransmitter itself, through the intracellular chloride concentration [96]. Conversely, chloride influx through GABA_A_Rs can modulate the reversal potential of GABA_B_R GIRK-mediated IPSPs, thus reducing their magnitude [97]. Moreover, the level of GABA_B_R activation can affect the tonic GABA_A_R-mediated inhibition by controlling the subunit composition of GABA_A_Rs mediating tonic inhibition [98,99]. The inhibition is regulated in a local manner across various dendrites, and it is dynamically regulated by activity and extracellular GABA levels in a homeostatic manner [100]. Moreover, the regulation of the GABA_B_R function can also happen on a cell-subtype specific level, with different effects on different interneuron types. For example, in the hippocampus, the activation of GABA_B_Rs modulates GABA release from PV interneurons with significantly less efficacy than from other types of interneurons [101].

During early life, the combination of glutamatergic and GABAergic activity can lead to large synchronous activity, which can be observed in in vitro preparations of rodent immature hippocampus as large synchronous network discharges called giant depolarizing potentials (GDPs). They influence synaptic transmission and circuit formation [102], and they lead to the release of large quantities of GABA in the extracellular space, which is sufficient to activate GABA_B_Rs [103]. Thus, GABA_B_R activation influences the duration of GDPs by promoting their termination [67,104]. 

In summary, the ability of GABA_B_R to modulate both excitatory and inhibitory synaptic transmission, as well as cell excitability via tonic GABAergic inhibition, can influence early synchronous activity in the developing brain.

## 6. Circuit Mechanisms

The development of neuronal circuits in the brain is characterized by the emergence of specific activity patterns that reflect the maturation state, and it is influenced by thalamic as well as local activity [105]. GABA_B_Rs have been shown to be able to influence network activity [106] and its entrainment to specific frequencies [107] in the hippocampus. In the thalamus, thalamocortical relay neurons and thalamic reticular neurons, which contribute to the generation of thalamic rhythmic activity, express both GABA_B_Rs and KCTD16. The strength and the frequency of the oscillatory behavior is controlled by GABA_B_Rs [108]. In the cortex, persistent brain activity requires a precise and a dynamic control of the balance between excitation and inhibition [109]. The synchronous activation of a large number of cells during a network oscillation and the consequent elevated release of GABA is sufficient for the activation of extrasynaptic GABA_B_Rs in a relatively large volume. Indeed, GABA_B_Rs are involved in the termination of the state of synchronous network firing (UP states) [110]. In the medial entorhinal cortex of rats, the termination of the UP state can happen either through a spontaneous mechanism, which is mediated by activation of GABA_B1a_-containing GABA_B_Rs, or through the activation of layer 1, which requires GABA_B1b_-containing GABA_B_Rs [111]. Thus, GABA_B_R activation can increase the variability of the oscillatory cycle, thereby having a desynchronizing effect on network activity, as opposed to the activation of GABA_A_Rs [112]. A lack of GABA_B_R activation leads instead to a decrease in the complexity of brain activity [113].

The layer 1-dependent termination of the UP state is mediated by the release of GABA from neurogliaform cells (NGFCs), which are known to use volume transmission for the inhibition of a large number of target cells [114]. Thalamic activation of NGFCs can occur in a coordinated manner across the neocortex, thus providing synchronization of different brain areas for transition to the regime of low network firing (DOWN state), as happens in slow wave sleep [115]. The NGFCs play an important role in defining the length of the integration window of sensory inputs to the cortex by reducing the thalamic feedforward inhibition in layer 4 through GABA_B_Rs [116]. 

The complexity of GABA_B_R actions can also result from input-specific mechanisms. As an example, in the piriform cortex superficial layers, two subtypes of glutamatergic cells, semilunar cells, and superficial pyramidal cells, receive inputs from the olfactory bulb. In addition, the latter population receives inputs from the former and from other brain areas. The activation of GABA_B_Rs can simultaneously decrease the excitability both post-synaptically in glutamatergic neurons and pre-synaptically in input terminals, providing disinhibition by reducing GABA release. A strong activation of GABA_B_Rs induces a biphasic response, consisting of inhibition followed by network excitation. This effect can be explained by the fact that superficial pyramidal cells display a stronger effect of GABA_B_Rs activation on pre-synaptic inhibitory terminals [117]. The anterior piriform cortex, together with the olfactory nucleus, in turn projects a feedback connection to the olfactory bulb, which is also controlled by GABA_B_Rs in an input-specific manner. Pre-synaptic GABA_B_Rs depress the inputs to the interneurons resident in the olfactory bulb, but not on the principal cells, thus their activation leads to a decrease in feedback inhibition on excitatory cells [118]. 

The strength of GABA_B_R-mediated inhibition can also be modulated by activity [119] and by the emergence of sensory activity. The medial superior olive (MSO) is a nucleus in the auditory brainstem responsible for detecting sound direction based on interaural time difference. While before the hearing onset GABA_B_Rs mediate a strong inhibition of both excitatory and inhibitory inputs as well as post-synaptic inhibition, after the hearing onset only the inhibition of inhibitory inputs remains unchanged. The GABA_B_R-mediated presynaptic suppression of excitatory inputs disappears over some weeks as well as the activation of GIRK-mediated currents in the post-synaptic side. Immunohistochemistry revealed how across the first month, the distribution of GABA_B_Rs in the MSO switches from mostly dendritic to prevalently somatic, mirroring the functional change [120].

Plasticity mechanisms are strongly influenced by GABA_B_Rs. For example, in the auditory cortex, transient activation of layer 4 neurons evokes a plastic response measurable 1 h afterwards, which consists of a specific strengthening of low gamma oscillations. This is achieved by both the enhancement and the suppression of the firing rate of individual cells in a layer-specific manner; GABA_B_Rs mediate both the strengthening of excitatory synapses from layer 4 to superficial layers and the inhibitory plasticity between layer 4 and layer 5. Thus, GABA_B_R modulation of network activity can influence the sensory adaptation to the presentation of a repeated stimulus and the sharpening of the cortical output [121]. The role of GABA_B_Rs in modulating inhibitory inputs at pyramidal cells in the auditory cortex is known to be dependent on sensory experience and the developmental stage [122]. In the same area, GABA_B_Rs are necessary for the generation of long-term depression on the connection between PV interneurons and pyramidal neurons, thus playing a crucial role in plasticity during the critical period of circuit development [123]. Similarly, GABA_B_R-mediated modulation could strongly influence the critical period for ocular dominance (OD) plasticity in cats in vivo. The activation of GABA_B_Rs could promote OD plasticity, and a blockade could prevent it. Interestingly, this effect showed a developmental pattern, as pharmacological manipulation of GABA_B_Rs did not affect OD plasticity in adult animals [124].

The effect of either activation or inhibition of GABA_B_Rs can therefore depend on the specific circuit being investigated, as they can be differentially expressed on specific cell types and vary during development. 

## 7. No Plan B: Dysfunction of GABA_B_R-Mediated Inhibition in Pathology

Given the many processes GABA_B_Rs take part in, it is not surprising that reduced or absent GABA_B_R function in mice has severe consequences. Lack of either of main subunits GABA_B1_ or GABA_B2_ leads to the development of seizures, which may cause death by the end of the first postnatal month [125,126], and to the development of hyperlocomotor activity, hyperalgesia and memory deficits [125,126,127]. Interestingly, mice lacking GABA_B1a_ display impairments in hippocampal synaptic plasticity and memory that could not be detected in GABA_B1b_ KO mice [23]. Furthermore, alteration of the GABA_B_R system has been observed in several pathological conditions and can contribute to the manifestation of epilepsy and psychiatric disorders [11,128].

### 7.1. Epilepsy

While GABA_B_Rs may contribute to epileptogenesis, they traditionally received less attention than GABA_A_Rs in this context [129]. Analysis of tissue from temporal lobe epilepsy patients revealed alterations in the hippocampal levels of GABA_B_Rs [130], and a reduction in GABA_B_R function was reported in the cortex of a rat model of absence epilepsy [131] and in human temporal lobe epilepsy tissue [132]. A possible mechanism of participation of GABA_B_Rs in seizure generation is via affecting the excitation to inhibition balance. Synapsin triple KO is a mouse model that generally displays alterations of GABAergic activity followed by epilepsy. In this model, reduced GABA release leads selectively to a weakening of GABA_B_R-mediated presynaptic inhibition of glutamate release, thus shifting the excitation to inhibition (E/I) ratio towards excitation [133]. 

Another example is cortical dysplasia, which is often associated with non-pharmacologically tractable seizures. In vitro application of 4-aminopyridine, a blocker of K+ channels, to human tissue with different types of cortical dysplasia can lead to the generation of spontaneous discharges. It was recently shown how the activation of GABA_B_Rs can maintain the network in a less susceptible state, since GABA_B_R blockade is required to induce ictal discharges [134]. However, the effects of the modulation of GABA_B_R activity can be contextual. Cyclin dependent kinase like 5 (CDKL5) KO mice, a mouse model of CDKL5 deficiency disorder, exhibit seizures in early life, as well as intellectual disability later during development. In the perirhinal cortex of CDKL5 KO mice, the number of inhibitory synapses is increased compared to control animals, and long-term potentiation (LTP) is reduced. The blockade of GABA_B_Rs, but not of GABA_A_Rs, could rescue the morphological changes and the memory deficits in vivo, indicating that not only reduced but also enhanced GABA_B_R activity can have a detrimental effect [135]. 

### 7.2. Autism Spectrum Disorders

Epilepsy is not the only pathological state in which GABA_B_R-mediated inhibition plays a crucial role. Postmortem tissue from individuals with autistic spectrum disorders (ASD) display a reduced GABA_B_Rs expression [136,137]. The evaluation of the role of GABA_B_Rs in ASD involves investigation of possible underlying mechanisms in a range of heterogeneous mouse models. Fragile X syndrome (FXS) is a neurodevelopmental syndrome which is often associated with intellectual disability, ASD, and epilepsy. Fmr1-KO mice recapitulate some of the features observed in patients. In this model, a selective decrease in GABA_B1a_ expression was observed in the hippocampus, accompanied by a reduced pre-synaptic inhibition of glutamatergic transmission. No changes could be seen on the inhibition of GABAergic inputs, leading to an imbalance in the E/I ratio, which could be rescued by administration of baclofen, a GABA_B_R agonist [137]. However, using younger animals, a different group reported a pathway-specific disruption in feedforward inhibition in the hippocampus [138]. Those mice displayed increased power in high gamma activity, as measured by EEG. Treatment with baclofen normalized the aberrant activity as well as a subset of deficits, including sensory processing and working memory but not social interaction [139]. Indeed, the effects of baclofen on the social behavior of Fmrp1-KO mice has been mixed, and it may potentially depend on additional factors such as dose, animal stress level, or experimental paradigm [139,140,141,142]. A recent study in Fmr1-KO mice in the medial prefrontal cortex highlighted that the pattern of differences depends on the developmental stage; animals between P14 and P21 displayed an increased inhibitory drive, but between P36 and P42 the picture was instead reversed [143].

Similar developmental differences were found in the medial prefrontal cortex of a mouse model of tuberous sclerosis. Tuberous sclerosis is a monogenic syndrome with an elevated association with ASD, epilepsy, intellectual deficits, and alterations in synaptic transmission [144]. In Tsc2^+/−^ mice, tonic GABA_B_R-mediated inhibition on layer 2/3 pyramidal neurons is reduced at P25–30 but not earlier, and it is accompanied by increased excitability [145]. Interestingly, an increase in the E/I ratio is observable between P15 and P19, but only transiently, since the early increase in glutamatergic synaptic transmission is followed by a matched potentiation in inhibitory transmission as compared to control animals. [145]. Interestingly, the effect of baclofen on pre-synaptic GABA_B_Rs was found to be comparable at glutamatergic synapses but increased at GABAergic synapses, thus leading to a baclofen-mediated shift in the E/I ratio toward excitation [146].

Another protein that was shown to associate with GABA_B_Rs is PIANP [45]. The PIANP KO mice displayed a behavioral phenotype similar to ASD mice models, featuring increased anxiety, repetitive behavior, reduced explorative behavior, and abnormal social behavior [147]. In those mice, the effect of baclofen on mEPSC and mIPSC frequency was reduced, as well as the effect on high frequency stimulation, highlighting how GABA_B_Rs can potentially underlie some of the manifestations.

### 7.3. Alzheimer’s Disease

Not only neurodevelopmental, but also neurodegenerative disorders have been associated with alterations in GABA_B_Rs. For example, GABA_B(1a,2)_ receptors have been shown to interact with APP through the sushi domains [45] and a decrease of the GABA_B_R number has been shown in Alzheimer’s disease (AD) patients [148] and in AD animal models [149]. Only recently, a possible mechanism of GABA_B_R involvement in this disease has been put forward. The interaction of GABA_B(1a,2)_ with APP has the double effect of stabilizing GABA_B_Rs on the axonal cell surface and of preventing the cleavage of APP into Aβ. Therefore, stabilizing pre-synaptic GABA_B_Rs in AD could lead to both the reduction of increased glutamatergic transmission and the secretion of Aβ [150]. Furthermore, activation of GABA_B_Rs with baclofen in AD rats led to the stimulation of the PI3K/Akt pathway, as well as the rescue of the hippocampal atrophy and apoptosis levels [151]. Generally, modulation of GABA_B_R activity has been shown to be able to lead to beneficial effects on cognition, learning, and spatial memory in the context of AD and dementia [151,152,153].

### 7.4. Long-Term Effects

Given the important role of GABA_B_Rs in development, it is reasonable to suggest that alterations in a specific time window, even if compensated later, may nevertheless lead to long-term consequences. The reduced functionality of GABA_B_Rs in the hippocampus may represent a consequence of epileptic seizures during early life. Epileptiform activity impairs GABA_B_R-dependent pre-synaptic inhibition of GABAergic terminals [154], and it may have long term effects [155]. A single dose of GABA_B_R antagonist administered at P15 could induce seizures that originate in the hippocampus and that have long-lasting effects, such as a decrease in paired pulse inhibition in CA1, which was measurable at P44 [156]. Similarly, early life systemic inflammation, as induced by a single injection of lipopolysaccharide at P14, leads to a reduced seizure threshold in P60 animals but not in those at P40. Concomitantly, it leads to a reduction in GABA_B_R-mediated inhibition and a subsequent increase in the release probability at CA1 hippocampal synapses [157]. An increase in the baseline activation of GABA_B_Rs can have an effect on the behavior of the animals, for example, transient activation of GABA_B_Rs via daily injection of baclofen in mice between P14 and P28 led to the development of anxiety behavior in adult mice, which was tested between P60 and P80 [158]. The mechanism for such long-lasting effects might involve protein expression, circuit formation, or other unknown factors [66,159].

### 7.5. Stress

Long-term GABA_B_R-dependent changes can also be triggered by other factors, such as stressors. Chronic stress affects GABA_B_R function both pre- and post-synaptically in the hypothalamic periventricular nucleus, thus affecting the function of the hypothalamus–pituitary–adrenal axis [160]. Psychological stress leads to changes in GABA_B_Rs function in the prefrontal cortex in a cell-type specific manner, increasing the depression on parvalbumin interneurons and instead reducing that on somatostatin interneurons [161]. Interestingly, a lack of the GABA_B1b_ subunit fosters resilience, while a lack of GABA_B1a_ instead leads to increased susceptibility to anhedonia and social withdrawal following stress [162]. The involved mechanisms are still elusive, but it is hypothesized that the responsible circuit involves the ventral tegmental area-nucleus accumbens pathway, the dorsal raphe nucleus, and the hippocampus, and potentially adult neurogenesis and the serotoninergic system [163]. A recent study described how KCTD12 in the dentate gyrus can bidirectionally modulate the response to chronic social defeat stress in mice. Overexpression of KCTD12 in the dentate gyrus increased stress vulnerability, while downregulation could reverse stress-induced social avoidance [164], indicating that auxiliary proteins may play a crucial role in modulation of GABA_B_R-mediated effects. Furthermore, activation of GABA_B_Rs in the nucleus accumbens can improve spatial memory in stress-exposed rats [165]. 

To sum up, GABA_B_R activity has been shown to play an important role in the manifestation of various neuropsychiatric symptoms, including developmental and degenerative disorders. Alteration of their function may not manifest as a stable picture, but rather a dynamical rearrangement, especially during early development. Transient disturbances to their functionality in early life can lead to long-term consequences.

## 8. Pharmacological Modulation and Therapeutical Perspectives

In light of the previously discussed relevance of GABA_B_Rs in various pathological conditions, they represent a natural target for providing therapeutical support. Modulation of the GABA_B_Rs function has been taken into consideration for a wide range of disorders, such as depression [166], anxiety and mood disorders [167], substance use disorders [168], chronic pain [169], schizophrenia [170], and potential pro-cognitive aims [171]. 

### 8.1. Orthosteric Modulation

Even if several substances are available for modulation of their activity, baclofen is the only substance which has received FDA approval. It is currently used for the treatment of spasticity, and in particular, its most active form is the R-(-)-baclofen enantiomer (arbaclofen, [172]). Although several antagonists of GABA_B_R have been routinely used in preclinical studies, only one entered clinical trials: CPG36742. Despite the fact that CPG36742 showed a potency to ameliorate AD symptoms in mild patient cases, further investigation was not pursued [173]. 

Activation of GABA_B_Rs has proven effective for the treatment of some of the symptoms in ASD mice models. For example, in the BTBR and C58 mice, baclofen treatment fixed stereotyped behavior and social interaction deficit, albeit it could not rescue every behavioral impairment [174]. In the 16p11.2 deletion mice—which in humans is characterized by intellectual disability, ASD, seizures, and anxiety—baclofen can rescue some cognitive deficits and social interaction [175]. Cntnap2 KO mice typically have impairments in behavior and auditory processing, which can be mostly remediated by treatment with R-baclofen [176]. A reduced NMDA receptor function is a common trait in schizophrenia, intellectual disability, and ASD. Mice with such phenotype display social and cognitive deficits, together with alterations in the EEG gamma band. Activation of GABA_B_Rs can rescue an altered E/I balance, gamma synchrony, and behavioral deficits following NMDAR hypofunction [140].

Arbaclofen is safe and well-tolerated in children and adolescents, and in an exploratory study it proved to be effective in several measures including the Aberrant Behavioral Checklist (ABC)—Irritability subscale and other social interaction measures [177]. A subsequent clinical trial failed to replicate the preliminary findings and to meet the expected effect on the ABC subscales, the primary outcome. Benefits were found on the Clinical Global Impression of Severity score, in which a subset of patients demonstrated strong improvement [178]. Arbaclofen has been shown in a recent clinical trial to have a positive effect as an adjuvant to risperidone in several subscales of ABC [179].

Baclofen treatment could improve impaired visual sensory processing in individuals with ASD; and, interestingly, it could impair visual processing in neurotypical subjects [180]. Currently, two randomized, double-blind, placebo-controlled studies are evaluating the effects of Arbaclofen on social function deficits in children and adolescents with ASD (NCT03887676 and NCT03682978).

Despite the efficiency of baclofen in preclinical studies in FXS (discussed in the previous section), where activation of GABA_B_Rs could rescue some aspects of the pathology, albeit with some controversy regarding the precise effect on social behavior [139,141,142], in a clinical trial for treatment of FXS arbaclofen failed to meet the primary outcome measure. It did, however, produce significant improvements on secondary measures [181].

### 8.2. Allosteric Modulation

As previously mentioned, GABA_B_Rs can interact with a variety of effectors, and they are embedded in many pathways. This makes orthosteric modulation prone to undesired consequences. Oral baclofen treatment in humans may cause dizziness, muscle weakness, sedation, nausea, fatigue [182], and it can less often lead to memory-related issues [183,184]. Therefore, a good amount of effort was devoted to the development of positive allosteric modulators (PAMs) of GABA_B_Rs. Introduced at the beginning of the 2000s, those compounds can influence the effect of GABA on GABA_B_Rs, but without complete activation of the receptor, therefore avoiding or minimizing potential side effects. The most well-known compounds are CGP7930 [185], GS39783 [186], and Rac-BHFF [187], which interact with the receptors by binding a pocket at the interface of the transmembrane domains of the active GABA_B1_/GABA_B2_ heterodimer [188]. What makes the use of positive allosteric modulation a valuable mechanism, besides the reduced amount of side effects [189], is their possible region- [190] as well as a pathway- and species-specificity [191], which could be leveraged for therapeutical advantage. Many PAMs have already shown positive effects on alcohol seeking behavior in animal models (e.g., [192]), and they are currently being investigated in clinical trials (see [193]). 

Using CGP7930 as a starting point, CLH304a was developed, which can act as a negative allosteric modulator (NAM) [194]. This drug has been suggested to bind the GABA_B2_ subunit in the transmembrane domain and inhibit GABA_B_Rs constitutive activation [195]. Very recently, another negative allosteric modulator named COR758 was described, which likely binds a site in the GABA_B1_ subunit. It could successfully modulate GABA_B_R activity in rat dopaminergic neurons [196]. However, especially for the NAMs, very little is known about in vivo responses and safety, and more investigation is required before considering a clinical use.

Another promising way of modulating the activity of GABA_B_Rs is through their interaction with the components of their complexes [45]. For example, Sereikaite and colleagues recently identified the binding epitope of the KCTD12 auxiliary proteins to GABA_B_Rs and via the use of deep mutational scan and an iterative screening procedure, they obtained peptides with a higher affinity to KCTD12 than GABA_B2_, which could reduce the interaction between the auxiliary protein and the receptor. This methodology does not only open the way for the study of the interaction of KCTDs and GABA_B_Rs, but it could be used to extend our knowledge of other GABA_B_R -interacting proteins [197]. Recent development in protein–protein interaction modulation has made huge progress, as reflected by the increasing amount of clinical trials using such modulators [198]. In the context of treating brain pathologies, however, it will be necessary to evaluate the bioavailability of potential modulators and their ability to cross the blood–brain barrier. 

## 9. Conclusions

This review highlights how, despite their apparent simplicity, GABA_B_Rs possess varied physiological effects. This property arises from the rich number of effector proteins they can affect as well as their precise position within a neural circuit. Much evidence points to the important role of GABA_B_Rs at early developmental stages, however, several details need to be further investigated. In the pathophysiological context, an increasing number of studies suggest that the temporal aspect should not be overlooked. In particular, it would be extremely useful to obtain a more precise description of the long-term effects of disturbances of GABA_B_R activity during early development and by which mechanisms they are exerted. Such a perspective would provide valuable information for the investigation of different disease models, and it would provide indications on which developmental stage would be more useful to investigate. Thus, GABA_B_Rs represent a suitable target for treating a plethora of conditions which feature a decreased GABA_B_R-mediated inhibition, or as a therapeutical tool to influence network activity. The recent description of GABA_B_R structure in an unprecedented level of detail will likely foster advancements in the pharmacological methods that will be available for the investigation of their function, as well as the development of novel treatments such as allosteric modulators that exhibit pathway and/or area selectivity and reduced side effects. Similarly, the development of technologies to modulate protein–protein interaction will allow the investigation of the possible outcomes caused by modifications of the GABA_B_R complex components. 

## Data Availability

Not applicable.

## Abbreviations

AC	adenylyl cyclase
AD	Alzheimer’s disease
APP	β-amyloid precursor protein
ASD	autism spectrum disorders
BDNF	brain-derived neurotrophic factor
FXS	fragile X syndrome
GABA	gamma aminobutyric acid
GABA_B_R	GABA_B_ receptor
GIRK	G protein-coupled inward rectifying K^+^ channel
PAM/NAM	positive/negative allosteric modulator
PV	parvalbumin
